# Identification of the Coding-Complete Genome Sequence of a Novel Cytorhabdovirus in Tilia cordata Showing Extensive Leaf Chloroses

**DOI:** 10.1128/mra.00052-23

**Published:** 2023-03-16

**Authors:** Kira Köpke, Artemis Rumbou, Susanne von Bargen, Carmen Büttner

**Affiliations:** a Division Phytomedicine, Thaer-Institute of Agricultural and Horticultural Sciences, Humboldt-Universität zu Berlin, Berlin, Germany; DOE Joint Genome Institute

## Abstract

Here, we report the coding-complete sequence (14,152 nucleotides [nt]) of a novel cytorhabdovirus detected in Tilia cordata and tentatively named cytorhabdovirus tiliae. The assumed genome organization is 3′-N-P-P3-M-G-p6-p6’-L-5′. The N gene encodes the putative nucleoprotein (59.1 kDa), P encodes the phosphoprotein (34.7 kDa), P3 encodes the movement protein (23.1 kDa), M encodes the matrix protein (23.1 kDa), G encodes a glycoprotein (64.4 kDa), and L encodes the viral RNA polymerase (247 kDa). P6 and P6’ are overlapping open reading frames (ORFs), which may encode gene products of 7.9 and 9.5 kDa, respectively, of unknown functions.

## ANNOUNCEMENT

Plant viruses can be introduced from tree nurseries into different ecosystems, spread within them, and invade neighboring ecosystems through their vectors. As the epidemiological, ecological, and financial consequences of virus-infected nursery trees cannot be estimated, tested virus-free planting material is therefore highly recommended.

**FIG 1:**
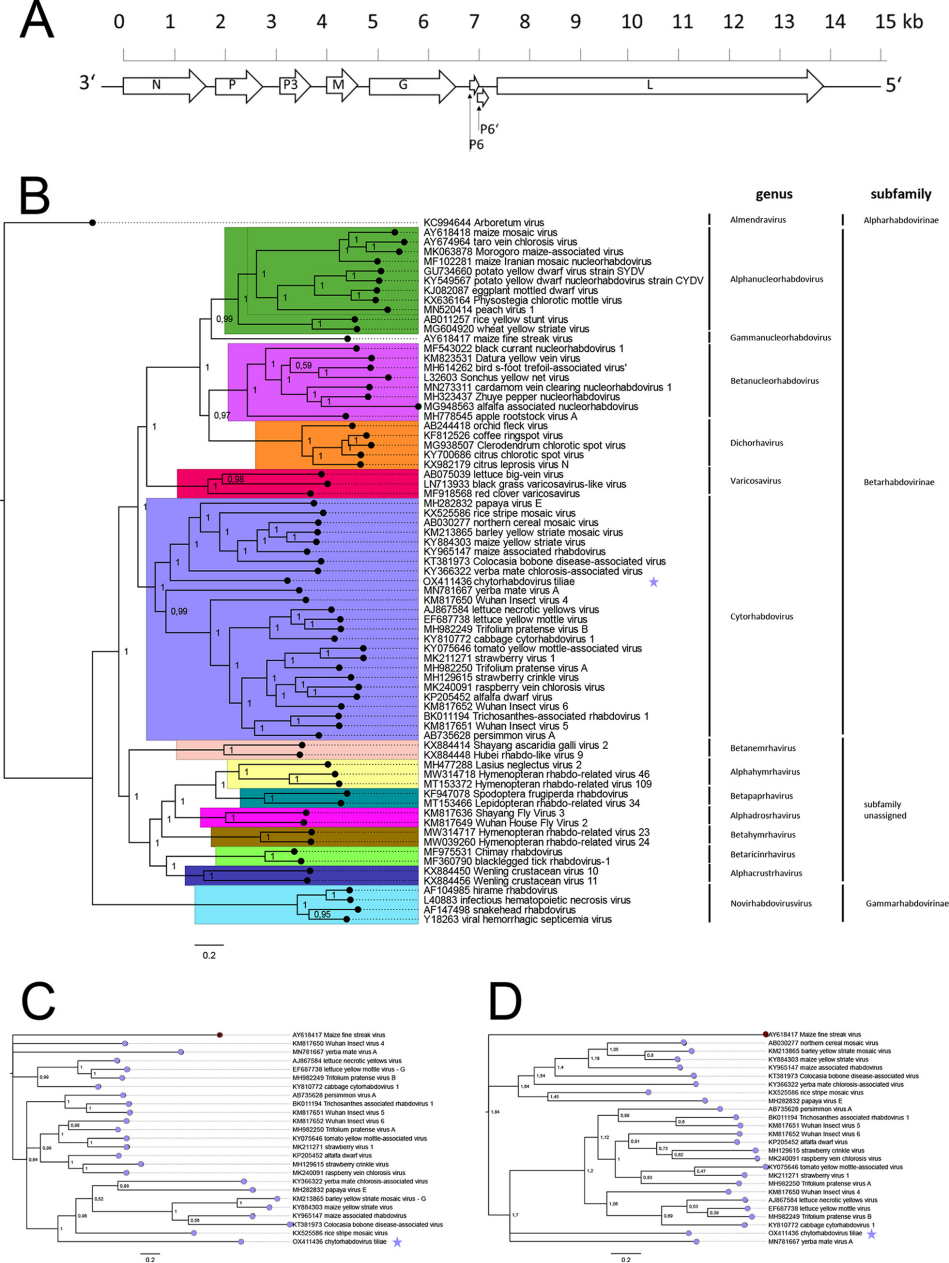
(A) Putative genome organization of the novel virus infecting *Tilia cordata*, tentatively named cytorhabdovirus tiliae. The assumed genome organization is 3′-N-P-P3-M-G-p6-p6’-L-5′. The N gene encodes the putative nucleoprotein, P encodes the phosphoprotein, P3 encodes the movement protein, M encodes the matrix protein, G encodes a glycoprotein, P6 and P6’are overlapping ORFs with accessory genes with unknown functions, and L encodes the viral RNA-polymerase. (B to D) Maximum clade credibility (MCC) trees inferred calculated by MRBAYES (7) from MAFFT v7.490 (8, 9) alignments of the novel cytorhabdovirus tiliae (marked with a star) and 72 full-length rhabdovirus ORF viral RNA-polymerase (L) nucleic acid sequences (B), 23 full-length cytorhabdovirus ORF glycoprotein (G) nucleic acid sequences (C), and 24 full-length cytorhabdovirus ORF nucleoprotein (G) nucleic acid sequences (D). As the outgroup, the sequences of the *Almendavirus* Aboretumvirus (A) or the *Gammanucleorhabdovirus* maize fine streak virus (C, D) were used. Branch lengths are drawn to scale with the scale bar showing the number of substitutions per site. Branches are labeled with posterior probabilities.

A leaf sample of a Tilia cordata tree showing chlorotic spots consistent with a viral infection was collected from a German tree nursery in 2016. Total RNAs were extracted from 0.3 g leaf tissue using the InviTrap spin plant RNA minikit (Stratec Molecular, Germany), followed by removal of remaining DNA with rDNase (Macherey-Nagel, Germany), and RNA purification using a NucleoSpin RNA clean-up kit (Macherey-Nagel). The rRNA depletion was performed using the RiboMinus plant kit for transcriptome sequencing (RNA-Seq) (Invitrogen, USA), followed by cDNA synthesis with the Maxima H minus double-stranded cDNA synthesis kit (Thermo Scientific) primed with random hexamers. Library preparation with the Nextera XT DNA library preparation kit (Illumina, USA), RNA sequencing (Illumina HiSeq 2500 platform; paired-end), and *de novo* assembly of contigs using BayersHammer (1) and SPAdes version 3.10 (2) were performed by BaseClear B.V. (Netherlands). As a second bioinformatics approach, we used GeneiousPrime 2022.1.1 (Dotmatics, USA) for data processing. The 774,739 reads, which had an average length of 115 bp, were trimmed using BBDUK 38.48 (3), with a minimum Phred score of 20, and we discarded short reads under 50 bp. Duplicate reads were discarded, and paired-end reads were paired by identifier and merged, if possible. Merged and unmerged paired reads were used for contig assembly using SPAdes assembler, version 3.10 (2). Default parameters were used except where otherwise noted. Eight overlapping contigs across both assemblies showing homologies to Cytorhabdovirus colocasiae (*Rhabdoviridae*, *Cytorhabdovirus*) were identified by BLASTx and tBLASTx (4) against the National Center for Biotechnology Information (NCBI) viral databases (accessed 15 February 2019) and were aligned by Geneious alignment into an unsegmented full coding virus genome (total size, 14,152 nt; mean coverage, 10.5×; GC content of 36.4%). The GeneiousPrime integrated open reading frame (ORF) finder was used to predict ORFs, and BLASTx (4) was used against the NCBI GenBank database and HHpred (5) for annotation. The novel cytorhabdovirus tiliae features the genome organization of Cytorhabdovirus and Nucleorhabdovirus with two accessory ORFs, namely, ORF 6 and an overlapping ORF 6’ with unknown function, between the G and L genes (Fig. 1A) (6, 7). To exclude assembly artefacts, the nucleic acid sequence between ORF G and L was verified by reverse transcriptase PCR (RT-PCR)-based amplification of this region followed by Sanger sequencing of amplicons (Macrogen Europe BV, Netherlands). Therefore, according to the manufacturer’s instructions, cDNA was synthesized using MaximaH minus reverse transcriptase (ThermoFisher Scientific, USA) followed by PCRs using Velocity DNA polymerase (Meridian Bioscience, USA) with an annealing temperature of 50°C and the primer combination Cy_Ti_G_F (5′-TGTTTCAGGTTTTGCAGG-3′) and Cy_Ti_6’_R (5′-GATTGGAGTAAGCAGCTC-3′) or Cy_Ti_6_F (5′-TCCGATTACAAAGATCCGAC-3′) and Cy_Ti_L_R (5′-GCCTTTTCTTGATATGAGCAG-3′).

Bayesian inference of phylogeny was performed by MRBAYES (8) from MAFFT version 7.490 (9, 10) alignments. The phylogenetic trees show that the novel virus presented here clusters in the clade of cytorhabdoviruses. (Fig. 1B to D). All assumed ORFs and ORFs of ICTV-approved cytorhabdoviruses were translated and aligned using Clustal Omega 1.2.2 (11). Alignments of amino acid sequences from the novel virus identified from *Tilia cordata* revealed identities of 12 to 22% (Table 1), which is far below the species demarcation criteria of less than 80% on an amino acid level of cognate gene products defined for cytorhabdoviruses by the ICTV (12), which is why we assume a new species. Further research is required to determine (i) the complete genome sequence of the novel cytorhabdovirus and (ii) the symptomatology, host range, geographic distribution, and impact of the virus on nursery-produced lime trees.

**TABLE 1:** Highest pairwise amino acid sequence identity values determined from aligned predicted ORFs of the novel cytorhabdovirus tiliae with corresponding amino acid sequences retrieved from GenBank^*a*^

Predicted ORF	Pairwise amino acid sequence identity (%)	Virus identity	Accession no.
N	21	Maize yellow striate virus	KY884303
P	12	Maize associated rhabdovirus	KY965147
		Yerba mate chlorosis-associated virus	KY366322
P3/MP	15	Northern cereal mosaic virus	AB030277
M	20	Maize associated rhabdovirus	KY965147
G	21	Maize yellow striate virus	KY884303
P6	20	Paper mulberry mosaic-associated virus	MN872813
P6'	18	Rose virus R	MT952336
L	22	Maize yellow striate virus	KY884303

a Accession numbers and virus names are indicated. Alignments were calculated using Clustal Omega1.2.2 (10). Cytorhabdovirus full-length ORF sequences were included in the data set from the following accession numbers: AB030277, AB735628, AJ867584, BK011194, BK014479, EF687738, KM213865, KM817650, KM817651, KM817652, KP205452, KT381973, KX525586, KY075646, KY366322, KY810772, KY884303, KY965147, MH129615, MH282832, MH982249, MH982250, MK211271, MK240091, MN781667, MN872813, MT381995, MT952336, and MW039593.

### Data availability.

The complete annotated genome sequence of the new cytorhabdovirus tentatively named cytorhabdovirus tiliae has been deposited in European Nucleotide Archive with the following numbers: OX411436 (CHROMOSOME_ACC), ERS14351145 (SAMPLE_ID), PRJEB57650 (STUDY_ID), and GCA_948193075 (ASSEMBLY_ACC). Sanger sequences has been deposited in GenBank with OQ361387 and OQ361388.
